# Peripheral Blood NRF2 Expression as a Biomarker in Human Health and Disease

**DOI:** 10.3390/antiox10010028

**Published:** 2020-12-30

**Authors:** Lee E. Neilson, Joseph F. Quinn, Nora E. Gray

**Affiliations:** 1 Department of Neurology, Oregon Health and Science University, Portland, OR 97239, USA; quinnj@ohsu.edu (J.F.Q.); grayn@ohsu.edu (N.E.G.); 2 Department of Neurology, Veterans Affairs Medical Center, Portland, OR 97239, USA

**Keywords:** Nuclear factor erythroid 2-related factor 2 (NRF2), Peripheral blood mononuclear cell (PBMC), biomarker, oxidative stress, antioxidant

## Abstract

Nuclear factor erythroid 2-related factor 2 (NRF2), a transcription factor which plays a critical role in maintenance of cellular redox, has been identified as a therapeutic target in a number of human diseases. Several reports have demonstrated beneficial effects of NRF2 manipulation in animal models of disease, and one NRF2-activating drug, dimethyl fumarate, is already approved for the treatment of multiple sclerosis. However, drug discovery is slowed due to a dearth of biomarkers which can inform target engagement and magnitude and duration of action. Peripheral blood mononuclear cells (PBMCs) are an accessible, minimally-invasive source of biomarkers which can be readily assayed and objectively monitored as a surrogate endpoint of NRF2 activation in clinical trials. We undertook a review of the literature on PBMC NRF2 measurements in human studies to explore its role as a suitable biomarker in various contexts of health and disease. It is clear that NRF2 and its target genes can be readily assayed from PBMCs in multiple disease contexts and may track with disease progression. Further work needs to be undertaken to evaluate its stability but should be considered as an exploratory marker in clinical trials targeting NRF2 activation.

## 1. Introduction

While reactive oxygen species (ROS) do serve as necessary signaling molecules, in excess, they can accumulate and drive organelle damage or even cell death. This process of oxidative stress is implicated in neurodegenerative disease [1], vascular disease [2], chronic obstructive pulmonary disease (COPD) [3], and many other conditions. One way to combat these deleterious free radicals is through activation of the endogenous antioxidant response pathway which drives increased expression of cytoprotective enzymes. The transcription factor nuclear factor erythroid 2-related factor 2 (NRF2, also called NFE2L2) is essential in regulating this pathway [4].

## 2. NRF2 is Regulated by Oxidative Stress, Inflammation, and Aging

**Figure 1:**
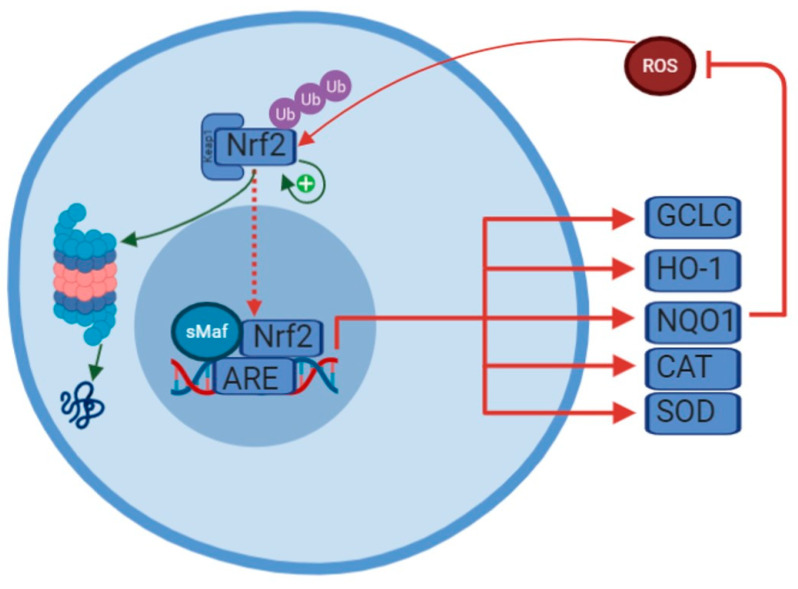
Regulation of NRF2. Normally, Keap1 targets NRF2 for ubiquitination and degradation by the proteasome. In response to oxidative stress, NRF2 is liberated from Keap1. NRF2 then translocates to the nucleus, heterodimerizes with small musculoaponeurotic fibrosarcoma (sMaf), and binds to the antioxidant response element (ARE). This is then able to drive transcription of a number of antioxidant genes. The ones mentioned in this review are highlighted here: NAD(P)H: quinone oxidoreductase 1 (NQO1), heme oxygenase 1 (HO-1), glutamate-cysteine ligase catalytic subunit (GCLC), catalase (CAT), superoxide dismutase (SOD). NRF2 has also been shown to activate its own promoter, thereby extending its duration of action.

Normally, NRF2 function is repressed though cytosolic binding with kelch-like ECH-associated protein 1 (Keap1), which tags NRF2 for ubiquitination and degradation by the proteasome [5,6] (Figure 1). However, in response to certain stimuli, including oxidative stress and inflammation, Keap1 is chemically modified at several cysteine residues which drives a conformational change such that binding to NRF2 and the subsequent ubiquitination is prevented [7]. Post-translational modifications of NRF2 itself, including acetylation and phosphorylation, can also disrupt its interaction with KEAP1 and increase nuclear translocation [8,9,10]. Additionally, NRF2 activity can also be controlled at the level of NRF2 transcription [11] and via autoregulation, through binding of ARE-like sequences in its own promoter [12]. The complete pathway is complex and there are undoubtedly other proteins which interact with this pathway. One such protein, DJ-1, which is ubiquitously expressed, is known to be deleterious when mutated as it is implicated in early-onset forms of Parkinson’s disease. It is known to oxidize itself in response to oxidative stress, thus acting in part as a buffer. It also interrupts the ubiquitination of NRF2 and chaperones it into the nucleus [13].

NRF2 expression does change across the lifespan, likely reflecting the phenomenon of “inflammaging”, the idea that aging is a chronic and low-grade inflammatory state [14]. It has been demonstrated that older animals have decreased nuclear content of NRF2 [15], and therefore functionally reduced NRF2 activity. Furthermore, NRF2 transcriptional upregulation in response to an acute bout of exercise is also blunted in aged individuals [11,16]. This demonstrates that aging affects NRF2 regulation at the levels of transcription, protein stability, and localization.

## 3. NRF2 Increases Antioxidant and Metabolic Gene Expression

When NRF2 is stabilized through its release from Keap1, it forms a heterodimer with the small musculoaponeurotic fibrosarcoma (sMaf) proteins, translocates to the nucleus, and binds to target genes [4] at the antioxidant response element (ARE) consensus sequence [17]. NRF2 binding regulates the transcription of enzymatic antioxidant defense proteins including glutathione (GSH; through de novo synthesis of glutamate-cysteine ligase, GCLC), heme oxygenase 1 (HO-1), NAD(P)H dehydrogenase quinone 1 (NQO1), catalase, superoxide dismutase (SOD), thioredoxin, and others. NRF2 has also been shown to regulate genes related to metabolic processes, including malic-enzyme 1 (ME-1), peroxisome proliferator-activated receptor γ (PPARγ), and transaldolase 1 which is reviewed in more detail in [18].

## 4. NRF2 Function is Important for Mitochondrial Function and Lifespan

NRF2-mediated transcription of these antioxidant and metabolism-related target genes also play a role in effective mitochondrial function. Hölmstrom et al. [19] showed that in cells where NRF2 was inactivated, mitochondrial function was impaired, as measured by a reduction in the mitochondrial membrane potential and reduced ATP synthesis. It has since been shown that increased NRF2 activity counterbalances mitochondrial ROS production, drives fatty acid oxidation, and supports mitochondrial integrity by promoting mitophagy [20]. It also seems to underlie normal neural stem cell self-renewal [21]. Interestingly, in a comparative biology study examining various rodent species, Lewis et al. [22] found that NRF2 activity positively correlated with maximum lifespan potential.

## 5. NRF2 Expression and Function is Changed in Several Disease Contexts

Since NRF2 has been demonstrated to be upstream of key mitochondrial, metabolic, and antioxidant pathways, several groups have used NRF2 expression as a readout of oxidative stress in a variety of disease contexts. In particular, studies in human subjects have employed measures of peripheral blood mononuclear cell (PBMC) NRF2 expression and activation [23] (Table 1). It is possible to assay mRNA, protein, and nuclear content of NRF2. Many groups have looked at downstream markers as well, such as NQO1 and HO-1. In each of the studies reviewed, the direction of change, if analyzed, is concordant between mRNA and protein.

### 5.1. Smoking and Chronic Obstructive Pulmonary Disease (COPD)

Cigarette smoking, a major risk factor of COPD, results in high levels of oxidative stress. Because NRF2 is known to be upregulated in response to oxidative stress, Garbin et al. [25] measured whether smoking results in increased NRF2 levels. They found that moderate smokers (5–10 cigarettes per day for 3 or more years) had a significantly elevated mRNA expression of NRF2 and its downstream target gene HO-1 in PBMCs compared to non-smokers. However, there were no significant differences in NRF2 or its downstream target genes in heavy smokers (25–40 cigarettes per day for 3 or more years) and controls [25]. Other studies also found a decrease in NRF2 in heavy smokers, while also demonstrating concordant decrease in HO-1 expression and an increase in oxidation products of PAPC (oxPAPC, a major component of cell membranes and lipoproteins which are known to trigger inflammatory responses) [26].

A similar pattern was observed in studies of COPD patients, where mRNA levels of NRF2 and its downstream targets GCLC and HO-1 were found to be elevated in COPD patients at baseline compared to controls [24]. However, after mean 40 months of follow up, Fratta Pasini et al. [27] found that expression of these factors was significantly reduced. The authors suggested that the early upregulation of NRF2 and its downstream target genes might represent a functional response to oxidative stress caused by smoking. The reduction in these factors later in disease might suggest a failure of these mechanisms to maintain this beneficial response in the context of exposure to chronic oxidative stress. Interestingly, in COPD patients that quit smoking, mRNA and protein expression of both NRF2 and HO-1 were again increased [24]. This suggests that patients may be able to re-initiate these beneficial anti-oxidant responses even later in disease through smoking cessation.

Because of the suggested beneficial effect of the NRF2-mediated antioxidant response, NRF2 activating drugs have been proposed for treatment of COPD. Pre-clinical studies utilizing a potent NRF2 activator, the triterpenoid CDDO, in mice exposed to chronic cigarette smoke significantly reduced oxidative stress and mitigated alveolar destruction, further supporting this idea [36]. Sulforaphane, a distinct NRF2 activator derived from cruciferous vegetables, was administered to patients with COPD in a double-blind, placebo-controlled fashion over six weeks [37]. However, this study failed to demonstrate target engagement, as NRF2 mRNA and expression of its target genes NQO1 and HO-1 was unchanged after treatment. This may be due to the short treatment paradigm, or the heterogeneity of the population studied. Thus, it remains untested whether NRF2 activation could improve clinical symptoms of COPD in human subjects.

### 5.2. End-Stage Renal Disease

Oxidative stress is also a common feature in renal disease, and Zaza et al. [29] were the first to evaluate changes in NRF2 expression in this context. They found significantly elevated mRNA and protein expression of NRF2 in PBMCs of peritoneal dialysis patients compared to healthy controls. In contrast, Pedruzzi et al. [28] later showed a marked reduction in expression of NRF2 mRNA and protein, and in its target gene NQO1 in PBMCs from patients on chronic hemodialysis. The opposing results in these two studies may be due to the stage of disease examined. The mean time on dialysis in the former study was 2.7 years, while the patient cohort on hemodialysis had been on this regimen a mean of 6 years. This fits with the data from COPD patients, where an initial rise, then fall in NRF2 levels was observed over the course of disease progression. The disparate results could also be due to the higher burden of stress and inflammation in the hemodialysis patients studied by Pedruzzi and colleagues, caused by its intermittent nature and dramatic swings in body volume and clearance of metabolic waste products compared to peritoneal dialysis.

Several studies in mice have demonstrated that manipulating NRF2 can modulate kidney function in the context of renal disease. NRF2 null mice have exaggerated and accelerated kidney injury, and conversely, NRF2 activating supplements have been shown to improve kidney function (as reviewed in [38]). Studies in patients with end stage renal disease have shown increased NRF2 expression in response to dietary interventions, including administration of starch-enriched cookies [39], and brazil nut supplementation [40], though these studies did not correlate these NRF2 expression changes with clinical measures. A third pilot study involving curcumin showed no change in NRF2 mRNA expression [41]. A series of clinical trials evaluating NRF2-activating drugs in chronic kidney disease patients have shown considerable promise and benefit [42,43]. This suggests that NRF2 activation may be beneficial in end-stage renal disease and is currently under study in phase 3 trials [44].

### 5.3. Metabolic Disorders

Diabetes mellitus (DM) type 2 has a complex pathophysiology, though oxidative stress has long been recognized to play a role and is closely associated with insulin-secreting beta cell dysfunction. PBMC NRF2 expression [30] and DNA binding activity [31] were both significantly reduced in patients with DM and poor glycemic control compared to healthy individuals. Subjects with insulin resistance were not found to have altered NRF2 levels [32], but those diagnosed with pre-diabetes already showed significant reductions in NRF2 activity [31], suggesting that changes in NRF2 expression already occur early in disease. Interestingly, those diabetics achieving euglycemia [31] did not exhibit changes in NRF2 activity, demonstrating that its function can be restored in DM upon achieving appropriate glycemic control. Camargo et al. [45] also found that dietary intervention could increase NRF2 levels in patients with metabolic syndrome, a disorder that often precedes development of DM.

While no clinical trials have been performed specifically aimed at increasing NRF2 levels in DM, it was found that administration of resveratrol significantly increased NRF2 expression in DM patients [46]. However, expression of NRF2 target genes HO-1 and catalase were not changed, and no meaningful clinical effects were reported. Therefore, it remains to be seen whether NRF2 activation could provide a beneficial effect for DM patients.

### 5.4. Coronary Artery Disease (CAD)

CAD is a chronic progressive disease of narrowing of the arteries which supply oxygen and nutrients to cardiac muscle and is directly related to endothelial dysfunction caused by oxidative stress [2]. When PBMC NRF2 mRNA and protein were evaluated in patients with stable CAD, it was found that their expression was reduced relative to healthy controls [35]. NRF2 target genes HO-1 and GCLC were also decreased. However, this was not replicated by more recent work, which found no change in NRF2 expression in PBMCs derived from CAD patients [34], though the authors speculate that comorbid DM and antioxidant medication use in their patient cohort may have confounded their results.

While no clinical trials have tested the use of NRF2 activating drugs in CAD patients, Turley et al. [47] did evaluate the effect of tert-butylhydroquinone (tBHQ), a commonly used food additive and known NRF2 activating substance, on NRF2 expression in vitro in the THP-1 monocyte cell line. Indeed, the authors found an increase in NRF2 expression in response to tBHQ treatment. This was also found to be the case when cells were pre-treated with serum derived from healthy controls. However, when cells were pre-treated with serum from CAD patients, tBHQ treatment no longer resulted in NRF2 upregulation. This suggests that there may be additional factors in CAD that actively suppress NRF2 expression, and may prevent its beneficial antioxidant effects as part of the pathophysiology of this disease.

### 5.5. Parkinson’s Disease (PD)

Parkinson’s disease, the second most common neurodegenerative disorder [48], centers on a defined motor syndrome—bradykinesia in combination with either rest tremor, rigidity, or both. It has a complex underlying pathogenesis, but it is well-established that excessive oxidative stress drives the neuroinflammation and cell death that underlie the disease [49]. NRF2 itself has been proposed to play a role in this disease process, as genetic haplotypes which result in increased NRF2 transcription are associated with delayed onset and overall decreased risk of PD [50]. Furthermore, elevated levels of urate, a known NRF2 activator, is protective in a familial form of PD [51]. There was also shown to be a positive correlation between NRF2 transcript levels in PBMCs and disease duration [33] in non-genetically defined PD patients. These studies all suggest that NRF2 expression and function are likely beneficial in the context of PD.

One study has found that NRF2 transcript and protein levels, and expression of its downstream target NQO1 are increased in PBMCs in PD patients of moderate severity relative to controls [33]. It will be interesting to determine in future work whether NRF2 levels decrease in late-stage disease, exhibiting a similar pattern in expression as in the diseases discussed above.

### 5.6. Multiple Sclerosis (MS)

MS is the most common chronic inflammatory disease of the central nervous system characterized by partially recovering episodes of acute demyelination which cause neurologic disability [52]. While a number of treatments are available to slow disease progression, it remains currently incurable. Dimethyl fumarate (DMF) has been FDA approved and seems to work through activating the NRF2-transcriptional pathway. Years after FDA approval, it was shown Nrf2 mRNA and NQO-1 were elevated in PBMCs, whereas HO-1 was not [53]. They also showed younger patients were able to mount a more robust increase in expression and, regardless of age, larger increases correlated with better clinical outcomes. This may represent one possible prognostic biomarker of drug efficacy, allowing more individualized drug decisions.

## 6. Potential NRF2 Activating Agents

Given the promise of NRF2 activation in the disease contexts discussed above, several studies have examined possible interventions to increase NRF2 expression or activation (Table 2). Similar to what was shown in vitro, when administered to healthy subjects, tBHQ was shown to increase expression of NRF2, as well as downstream target genes [47]. Known activators of the antioxidant response, curcumin [54], sulforaphane [55] and ozone [56] were also shown to increase NRF2 expression in healthy individuals. Similarly, exercise was found to acutely increase NRF2 activation, as indicated by an increase in the nuclear fraction of NRF2 and increased expression of its target genes HO-1 and NQO-1, though it did not change NRF2 mRNA levels [57]. However, Done and colleagues [57] found that this was only the case in younger individuals, suggesting that acute exercise may not be an effective means to activate NRF2 activity in diseases that affect older adults.

## 7. Discussion

NRF2, as a master regulator, is not specific to any one disease process, making it a poor tool for the diagnosis of any one disease. However, it might be broadly applied in contexts of increased oxidative stress as a biomarker of disease progression. One interpretation of the common findings in these studies is that NRF2 activation appears to be a normal, compensatory response to whatever insult is driving oxidative stress and then, at some point later in the disease process, antioxidant defenses become overwhelmed. One possible mechanism is through the ROS-mediated oxidation, and resultant degradation, of the DJ-1 protein which is essential for NRF2 protein stabilization [58]. This could explain the failure of some NRF2-activating interventions; if the population selected has exceeded antioxidant defense capacity, no amount of drug could engage the pathway and further drive the maximally-activated pathway. It will be important when translating this work, therefore, to enrich clinical trials with the appropriate population of patients whose antioxidant defenses have not been overwhelmed and therefore have the capacity for NRF2 activation.

It is also clear from these studies that NRF2 expression can be readily and reliably assayed from PBMCs. The actual technique employed in these studies were strikingly similar. They consistently excluded patients with inflammatory disorders and infectious diseases and those taking anti-inflammatory prescriptions and supplements. It is therefore unknown what role these medications and comorbidities might play. For example, several epidemiological studies have shown a protective effect in PD for those who take non-steroidal anti-inflammatory drugs (NSAIDs) (reviewed in [59]). Blood samples were almost consistently drawn in the morning and in the fasting state excepting the few instances of dietary interventions. It is unclear, then, if there are diet-based or circadian patterns to NRF2 expression. Nevertheless, when adhering to these restrictions, they are able to show clear differences between groups and show target engagement when applying an intervention. In the few studies which did so, there was concordance with PBMC NRF2 expression and local oxidative stress (e.g., in adipocytes in metabolic syndrome). It will be important for future studies to demonstrate if these peripheral changes reflect the same changes in target tissue.

While promising, it is important for these additional studies to be carried out, and larger cohorts to be followed longitudinally to validate this marker prospectively, before NRF2′s utility as a potential biomarker can be further clarified.

## 8. Conclusions

The antioxidant NRF2 pathway is a promising target for drug development in many disease states but its successful translation is limited by our current understanding of NRF2 biology. The data currently available from human PBMC studies show a characteristic pattern of increasing NRF2 levels early in the disease state and a decline as the disease progresses. While this is a useful general framework, it will be important to track these changes longitudinally to determine at what point NRF2 levels may normalize or decline and what external factors, such as diet, may influence them before being ready for widespread use.

## Figures and Tables

**Table 1:** Summary of observational studies evaluating Nrf2 expression in PBMCs from humans in different disease contexts. PRE = pre-diabetes; DM-C = controlled diabetes mellitus; DM-NC = uncontrolled diabetes mellitus.

Category	Population	Comparator	NRF2 mRNA Expression	NRF2 Protein Expression	Downstream Effectors	Other Markers	Reference
Pulmonary	Mild-moderate COPD, ex-smokers	Mild-moderate COPD, never smokers	Increased	Increased	Increased HO-1		[24]
	Moderate smokers Heavy smokers	Healthy Controls	Moderate—Increased Heavy—Decreased	Moderate—Increased Heavy—No change	Moderate—Increased HO-1 mRNA and protein Heavy—no change	NF-kB linearly correlated with smoking and inversely correlated with Nrf2	[25]
	Smokers	Healthy Controls	Decreased	Decreased	Decreased HO-1 and GCLC mRNA and protein expression		[26]
	COPD	Healthy Controls	Increased at baseline Decreased at 40 months		HO-1 and GCLC increased at baseline and decreased at 40 months		[27]
ESRD	Hemodialysis	Healthy Controls	Decreased		Decreased NqO1	Increased NF-kB	[28]
	Peritoneal dialysis	Healthy Controls	Increased	Increased			[29]
Metabolic	DM	Healthy Controls	Decreased	Decreased	Decreased HO-1		[30]
	PRE DM-C DM-NC	Healthy Controls		PRE—Decreased DM-C—Unchanged DM-NC—Decreased		DM—Glutathione and reduced glutathione decreased	[31]
	Obese Obese with insulin resistance	Healthy Controls	No change			No change in NF-kB	[32]
Neuropsychiatric	Parkinson Disease	Healthy Controls	Increased	Increased	Increased NQO-1, GCL, GR		[33]
Cardiac	CAD	Healthy Controls	No change		No change in NQO-1 mRNA	No change in NF-kB mRNA	[34]
	CAD	Healthy Controls	Decreased	Decreased	Decreased HO-1, GCLC		[35]

**Table 2:** Summary description of studies where a purported NRF2 activator was applied to humans, or human blood, and NRF2 expression was later examined in PBMCs.

Population	Intervention	NRF2 mRNA Expression	NRF2 Protein Expression	Downstream Effectors	Other Markers	Reference
Healthy subjects	Ozone every other day for 3 treatments v placebo	Increased 30 min after exposure and normalized after treatments			Decreased GSH	[56]
Healthy subjects	tert-butylhydroquinone (tBHQ), in vitro	Increased		Increased HO-1, NQO-1, GCLC	Decreased IL-2, IFN-gamma	[47]
Healthy subjects	PBMCs isolated and treated with acrolein +/− sulforaphane pre-treatment		Increased			[55]
Healthy subjects	PBMCs isolated and exposed to ionizing radiation +/− dendrosomal curcumin nanovehicle treatment	*Increased binding activity of Nrf2		Increased mRNA and protein expression of HO-1	Decreased activity NF-kB	[54]
Healthy subjects	Older (age 63) and younger (age 23) men assessed after single session of 30 min of exercise		Increased in whole cell, increased in nuclear fraction in young only at 4 h post-exercise	Increased HO-1 mRNA expression, no change in NQO-1 or SOD1		[57]
Metabolic syndrome	Randomized to 1 of 4 diets for 12 weeks each: (i) HSFA, (ii) high-monounsaturated fatty acid, and (iii), (iv) two low-fat, high-complex carbohydrate diets supplemented with n-3 polyunsaturated fatty acids or placebo	HSFA—Increased at 2 h, normalized at 4 h Ii, iii, iv—No change	HSFA—Increased nuclear fraction at 2 h, normalized at 4 h Ii, iii, iv—No change		HSFA—Increased mRNA expression of antioxidant defense-related genes SOD1, SOD2, CAT, GPx1, GPx4, GSR, TXN, andTXNRD1	[45]
Hemodialysis	Resistant starch-enriched cookies in 4-week crossover design	Increased		Increased NQO-1 protein expression		[39]
Hemodialysis	Curcumin v placebo for 12 weeks	No change			Decreased hsCRP, NF-kB mRNA expression	[41]
Hemodialysis	Brazil nut v placebo for 12 weeks	Increased		Increased NQO-1 mRNA	Decreased NF-kB mRNA expression, decreased IL-6	[40]
COPD	Placebo v. low-dose sulforaphane v. high-dose sulforaphane daily by mouth for four weeks	No change		No change in NQO-1 or HO-1		[37]

## Data Availability

No new data were created or analyzed in this study. Data sharing is not applicable to this article.
